# Supplementary material for: Sedimentary noise and sea levels linked to land–ocean water exchange and obliquity forcing
Source: Nat Commun. 2018 Mar 8;9:1004. doi: 10.1038/s41467-018-03454-y (PMC5843644; doi:10.1038/s41467-018-03454-y)
Supplement: Supplementary file 1 — Supplementary Information [file 41467_2018_3454_MOESM1_ESM.pdf]

Nature Communications

DOI: 10.1038/s41467-018-03454-y

Supplementary Information for

Sedimentary noise and sea levels linked to land-ocean water exchange and obliquity forcing

Mingsong Li*, Linda A. Hinnov, Chunju Huang* & James G. Ogg

* Correspondence and requests for materials should be addressed to M.L. (email: limingsonglms@gmail.com) or to C.H. (email: huangcj@cug.edu.cn)

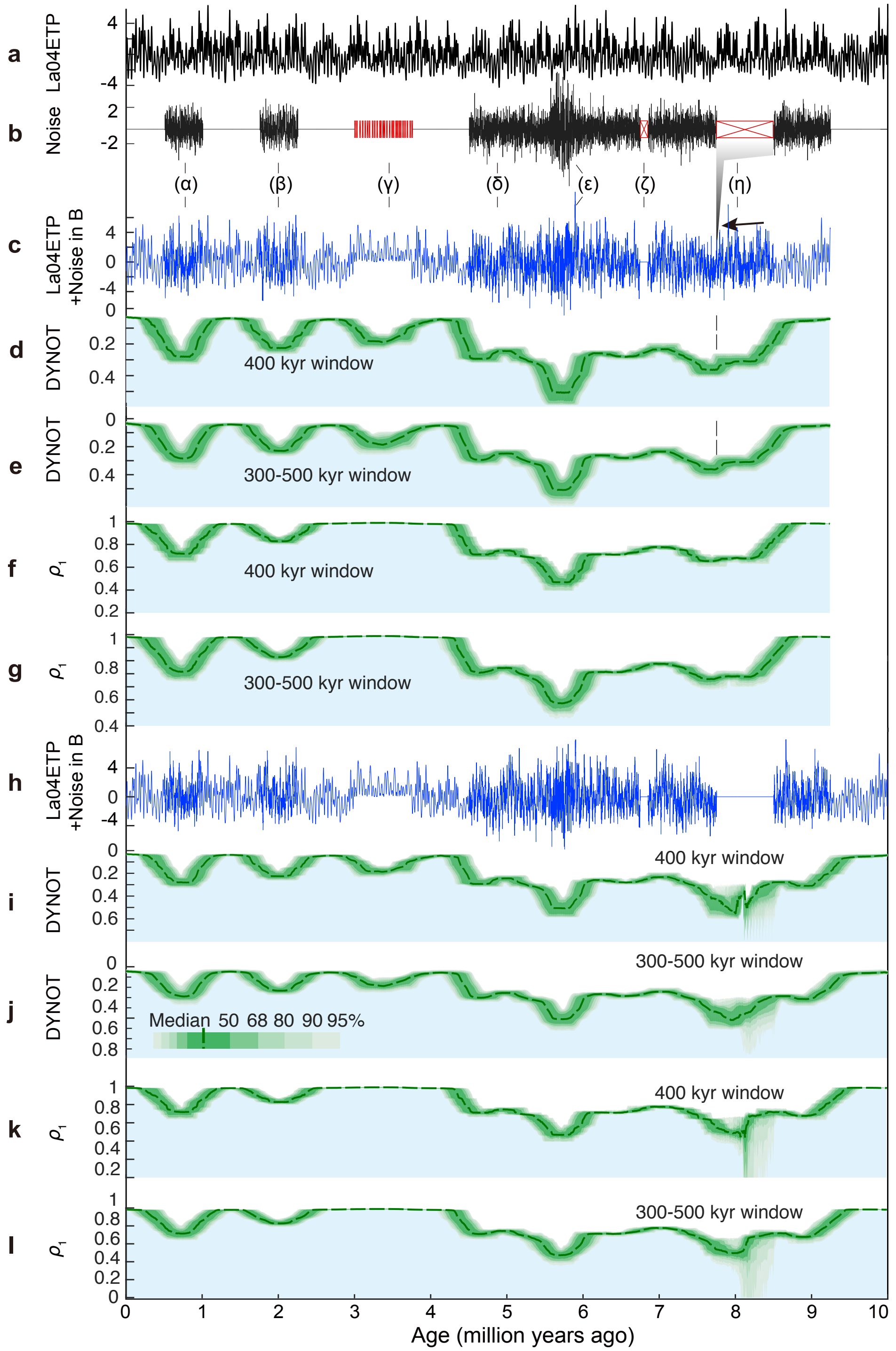

Supplementary Figure 1 Synthetic dynamic stratigraphic noise model of sea-level variations. **a** La2004 astronomical solution from 0 to 10 Ma (La04ETP, see Fig. 1 in the main text). **b** Seven intervals with different types of noise (black) and gaps (red). (α) 500-kyr white Gaussian noise. (β) 500-kyr red noise with a lag-1 autocorrelation coefficient 0.3. (γ) multiple intermittent, brief gaps. (δ) 4.75-myr white Gaussian noise with (ε) additional 500-kyr white Gaussian noise, (ζ) a 100-kyr gap, and (η) a 750-kyr gap. **c** Sum of La04ETP in (**a**) and the noise in (**b**). ETP series are expanded for "un-recognized" gaps (γ) or zero during the "recognized" gap (ζ) or missing during the "un-recognized" gap (η) in (**b**). **d-e** DYNOT model of series in (**c**). **f-g** ρ_1 model of series in (**c**). **h** Sum of La04ETP in (**a**) and the noise in (**b**). ETP series is expanded for "un-recognized" gaps (γ) or zero during the "recognized" gap (ζ and η) in (**b**). **i-j** DYNOT model of series in (**h**), curve minima correspond to noise intervals in (**b**). **k-l** ρ_1 model of series in (**h**). Confidence levels are estimated by a Monte Carlo analysis with 5,000 iterations using a running window of 400 kyr (**d**, **f**, **i** and **k**) or randomized windows within a 300-500 kyr range (**e**, **g**, **j** and **l**)

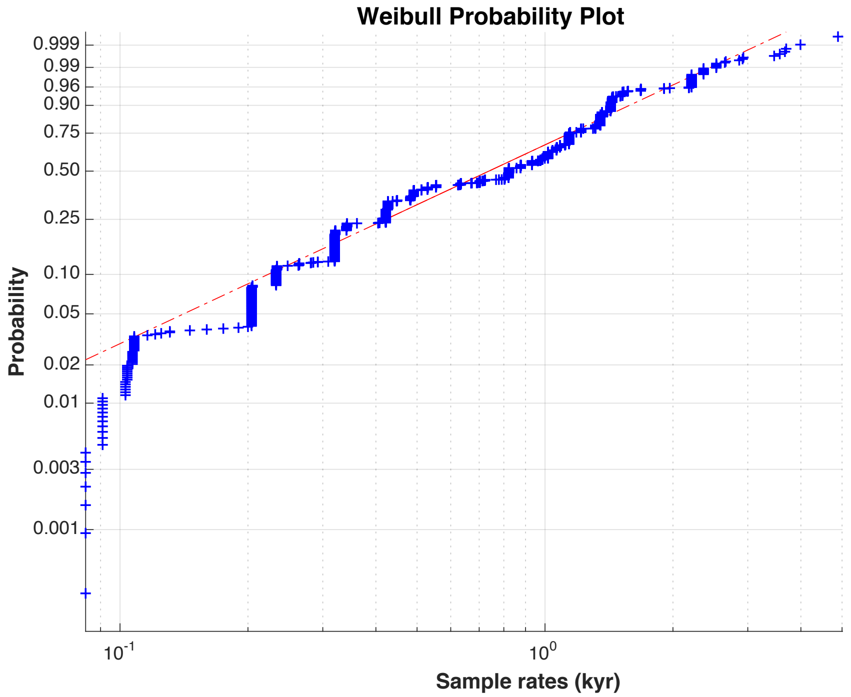

Supplementary Figure 2 The Weibull probability plot of sampling rates of the gamma ray series at ODP Site 1119 using Matlab's wblplot.m

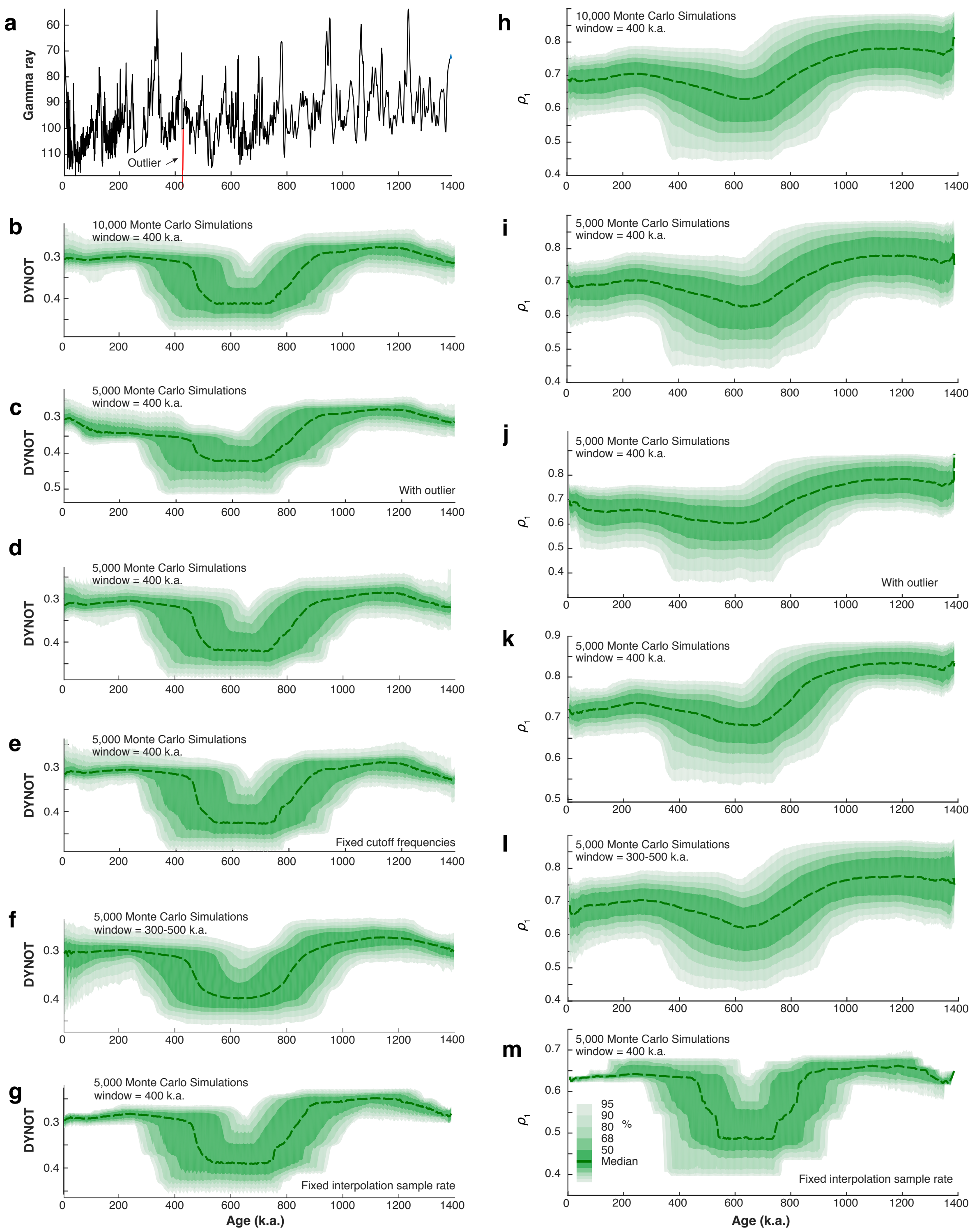

Supplementary Figure 3 Testing the DYNOT and ρ_1 models on the fine-tuned gamma ray log at ODP Site 1119 for the past 1.4 myr (Supplementary Figs. 4-5). **a** Gamma ray series is from ref. 1. **b-f** DYNOT models of interpolated time series in (**a**) using randomly selected sampling rates from 0.22 to 2.04 kyr with a running window of 400 kyr (**b-e**) and 300-500-kyr (**f**) and 10,000 (**b**), and 5,000 (**c-g**) Monte Carlo realizations. **g** DYNOT model with fixed interpolated sample rate of 1 kyr with a running window of 400 kyr and 5,000 Monte Carlo realizations. **h-m** ρ_1 model of interpolated gamma ray series using random sampling rates from 2.04 to 4.08 kyr (**h-j** and **l**) and 2.04-3.06 kyr (**k**) and a fixed sample rate of 4.08 kyr (**m**) with a running window range of 300-500 kyr (**l**), and a fixed window of 400 kyr (**h-k** and **m**), and 10,000 (**h**) and 5,000 (**i-m**) Monte Carlo realizations. Small differences between (**b**) and (**d**) indicate

5,000 Monte Carlo simulations are sufficient for the DYNOT and ρ_1 models (compare **h** and **i**). Dynamic noise of the gamma ray series with and without an outlier (red in **a**) using the same parameters suggest a significant outlier contribution to the noise spectra (compare **c** and **d**, and **i** and **j**). Small differences among **d**-**g** suggest that changes in sampling rates of original dataset, window selection (400 kyr or 300-500 kyr), and cutoff frequencies do not have a significant impact on the DYNOT model. This applies also to the ρ_1 model except that the ρ_1 model is sensitive to the sampling rate (**m**). 2π multi-taper power spectra are used to generate DYNOT models for all Monte Carlo realizations. Target astronomical cycles for all calculations have periods of 405 kyr, 125 kyr, 95 kyr, 40.9 kyr, 23.6 kyr, 22.3 kyr, and 19.1 kyr. Cutoff frequencies for estimating total variance are from 0.001 to 1 kyr^{-1}. All models are shown with median, 50%, 68%, 80%, 90%, and 95% ranges of the Monte Carlo simulations

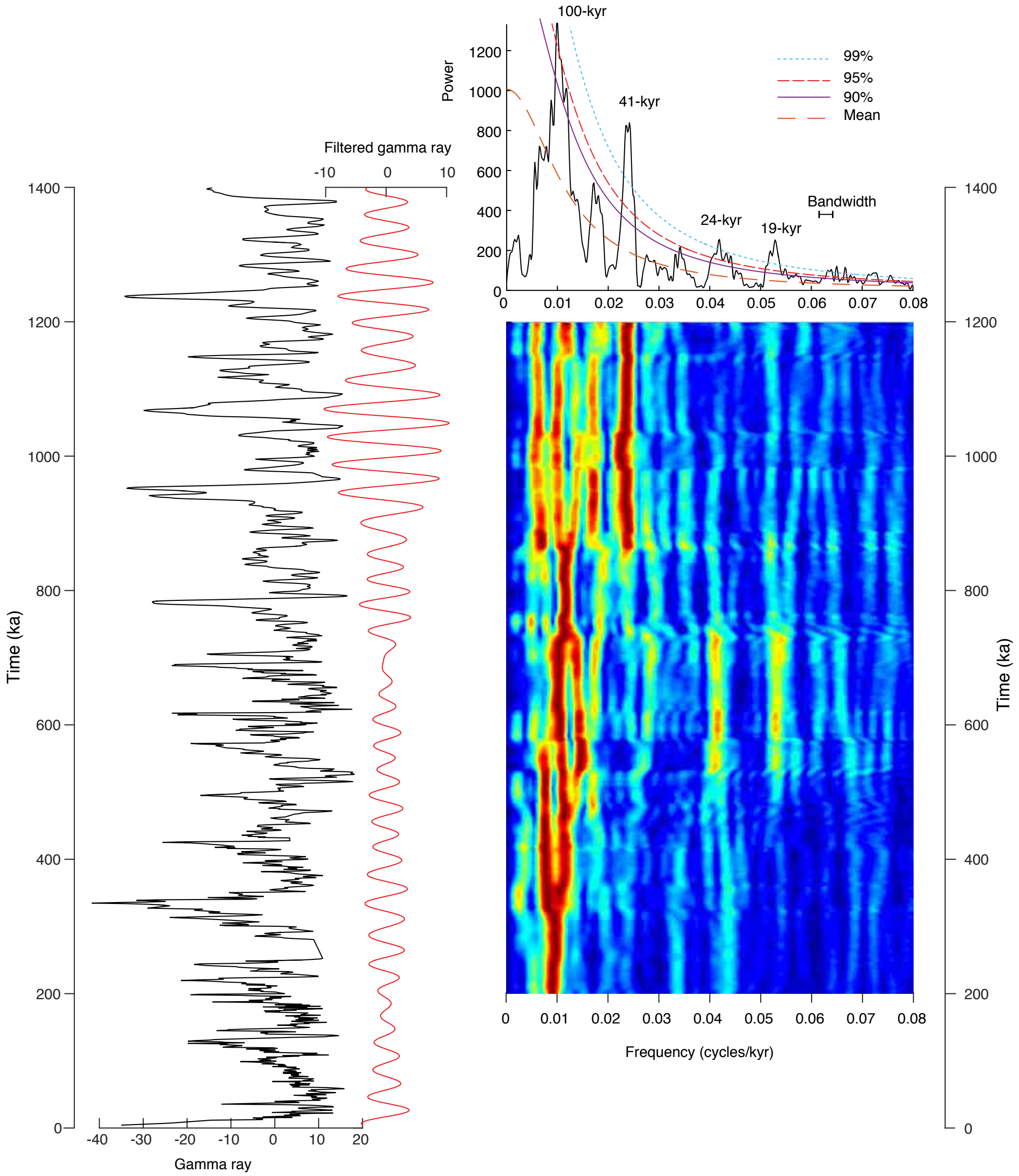

Supplementary Figure 4 Cyclostratigraphy of original gamma ray series at ODP Site 1119 from 0 to 1400 ka. Left: Original gamma ray series is from ref. 1 (black), and one outlier was removed (see Supplementary Fig. 3**a**), and an 800-kyr weighted average (LOESS) trend was removed. Obliquity cycles filtered from the gamma ray series (Gaussian filter, passband: 0.024 ± 0.004 kyr^{-1}). Right: 2π MTM power spectrum and evolutionary FFT of the gamma ray series using a 400 kyr running window

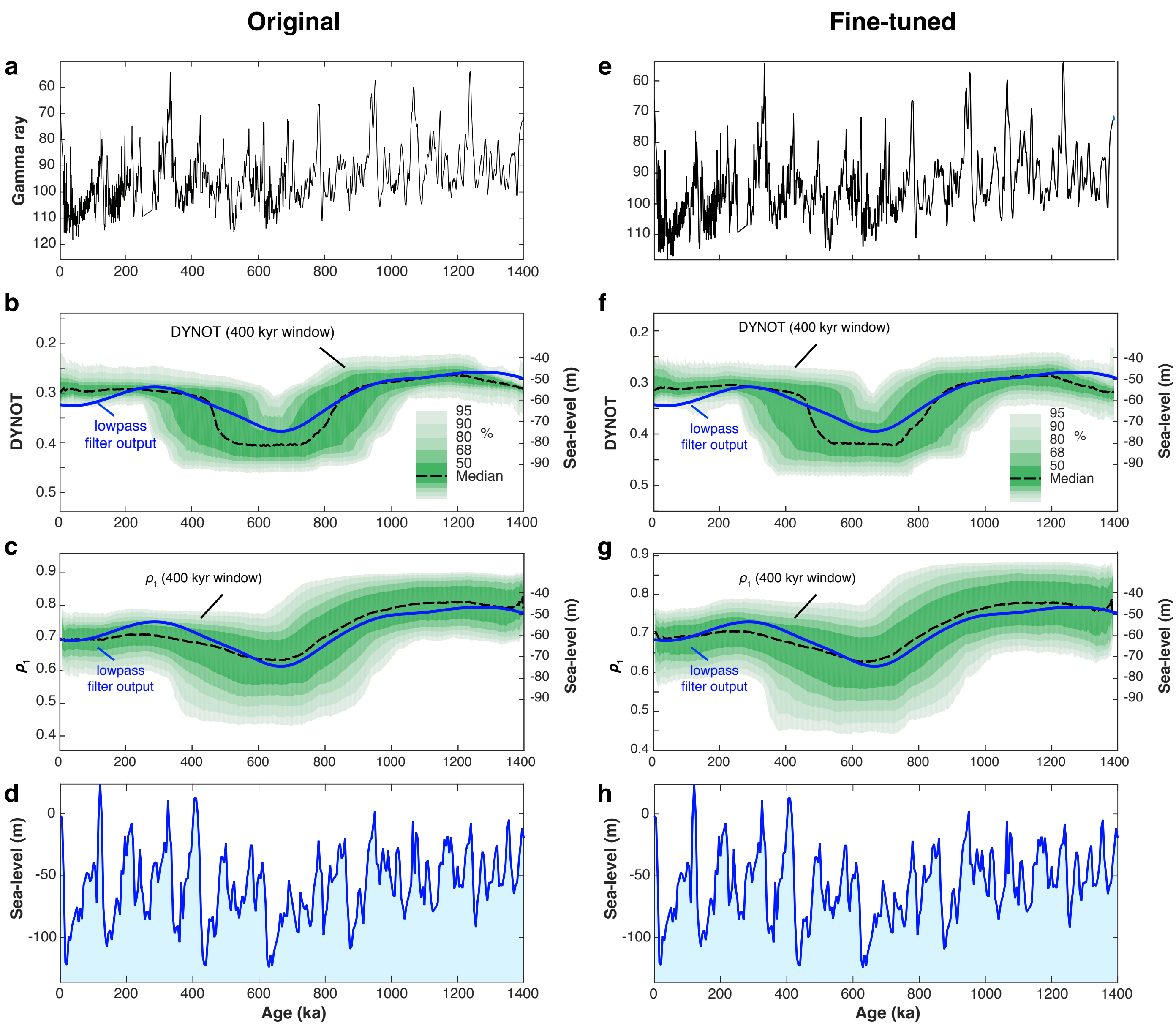

Supplementary Figure 5 Dating error and dynamic sedimentary noise models of the gamma ray log at ODP Site 1119. Left plots show results for original gamma ray time series, and right plots show results of fine-tuned gamma ray time series using the age model in Supplementary Table 3. Fine-tuning of gamma ray time series leads to only slight changes in the shape of the DYNOT model with a 400-kyr window (compare **b** and **f**), and little change in the ρ_1 model (compare **c** and **g**). **a** Original gamma ray time series with age model are from ref. 1, for which one outlier was removed (see Supplementary Fig. 3**a**). **b** DYNOT models of the interpolated time series in (**a**) using random sampling rates from 0.20 to 1.55 kyr with a running window of 400 kyr. **c** ρ_1 model of the time series in (**a**). **e** Fine-tuned gamma ray time series. **f** DYNOT models of interpolated time series in (**e**) using random sampling rates from 0.22 to 2.04 kyr with a running window of 400 kyr. **g** ρ_1 model of the time series in (**e**). Confidence intervals are estimated by Monte Carlo analysis with 5,000 iterations. (**c**) and (**g**) use random sampling rates of 2.04-4.08 kyr. The noise models are shown together with the lowpass Gaussian filter output (solid blue; cutoff frequency of 1/(400 kyr)) of (**d**, **h**) sea-level changes estimated from benthic foraminiferal $\delta^{18}O$ [2]

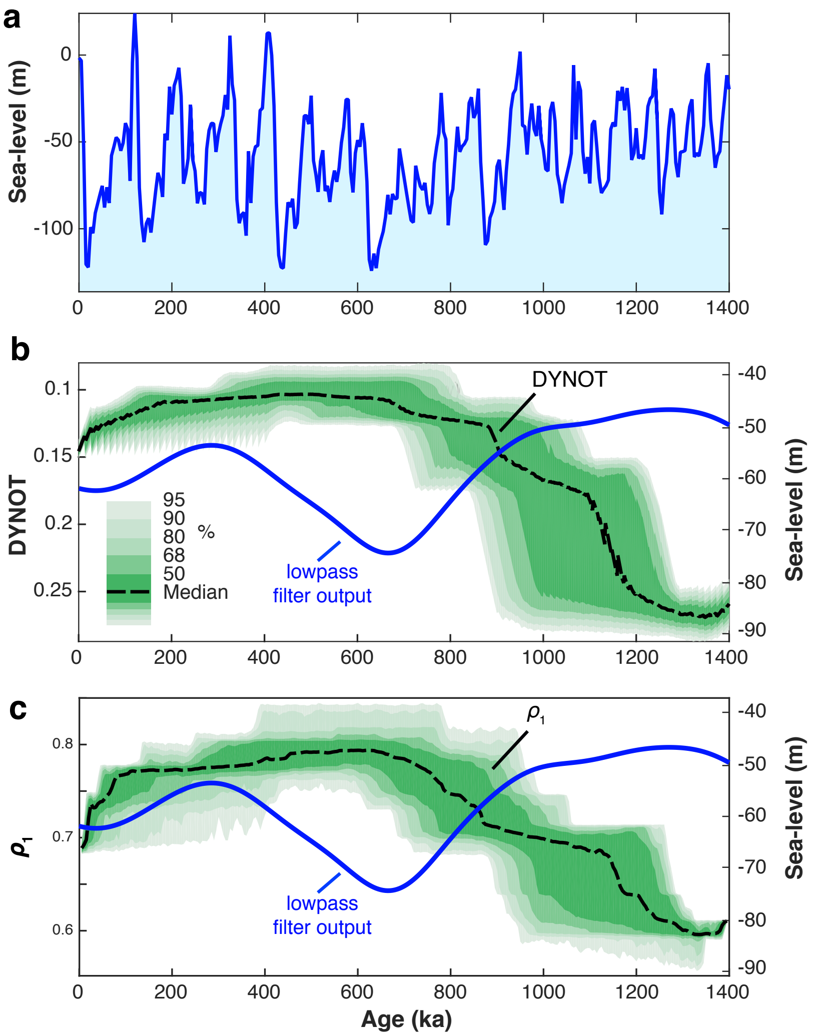

Supplementary Figure 6 The DYNOT and ρ_1 models of the global sea-level curve show little similarity to sea-level changes and dynamic sedimentary noises. Dynamic noise in the global sea-level curve is discussed in ref. 3. **a** Sea-level changes estimated from benthic foraminiferal $\delta^{18}O$ from 0-1400 ka [2]. DYNOT (**b**) and ρ_1 (**c**) models of the sea-level time series in (**a**) using a sampling rates of 5 kyr with a running window of 400 kyr. Confidence intervals are estimated by Monte Carlo analysis with 5,000 iterations. The models are shown with lowpass Gaussian filter output (solid blue; cutoff frequency is 1/(400 kyr)) for the sea-level time series in (**a**)

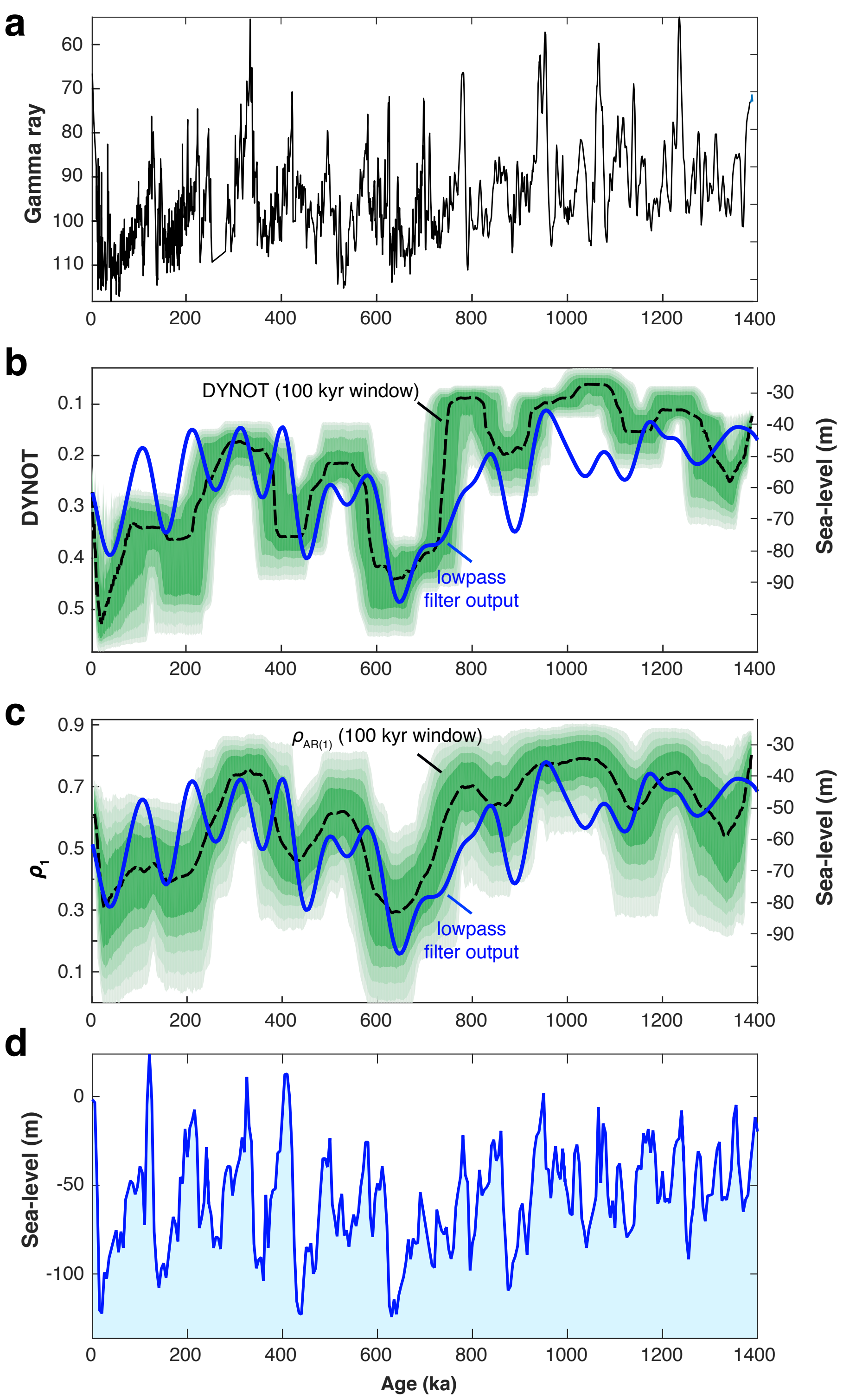

Supplementary Figure 7 Sedimentary noise models of the gamma ray log at ODP Site 1119 using a 100-kyr running window show higher frequency sea-level variations. **a** Fine-tuned gamma ray series, with one outlier removed (see Supplementary Fig. 3**a**). **b** DYNOT models of the interpolated time series in (**a**) using random sampling rates from 0.20 to 2.04 kyr with a running window of 100 kyr. **c** ρ_1 model of the time series in (**a**) use random sampling rates of 2.04-4.08 kyr. Confidence intervals are estimated by Monte Carlo analysis with 5,000 iterations. The noise models are shown with lowpass Gaussian filter outputs of sea-level changes (solid blue; cutoff frequencies are 1/(100 kyr)) of **d** sea-level changes estimated from benthic foraminiferal $\delta^{18}O$ [2]

Daxiakou

Chaohu

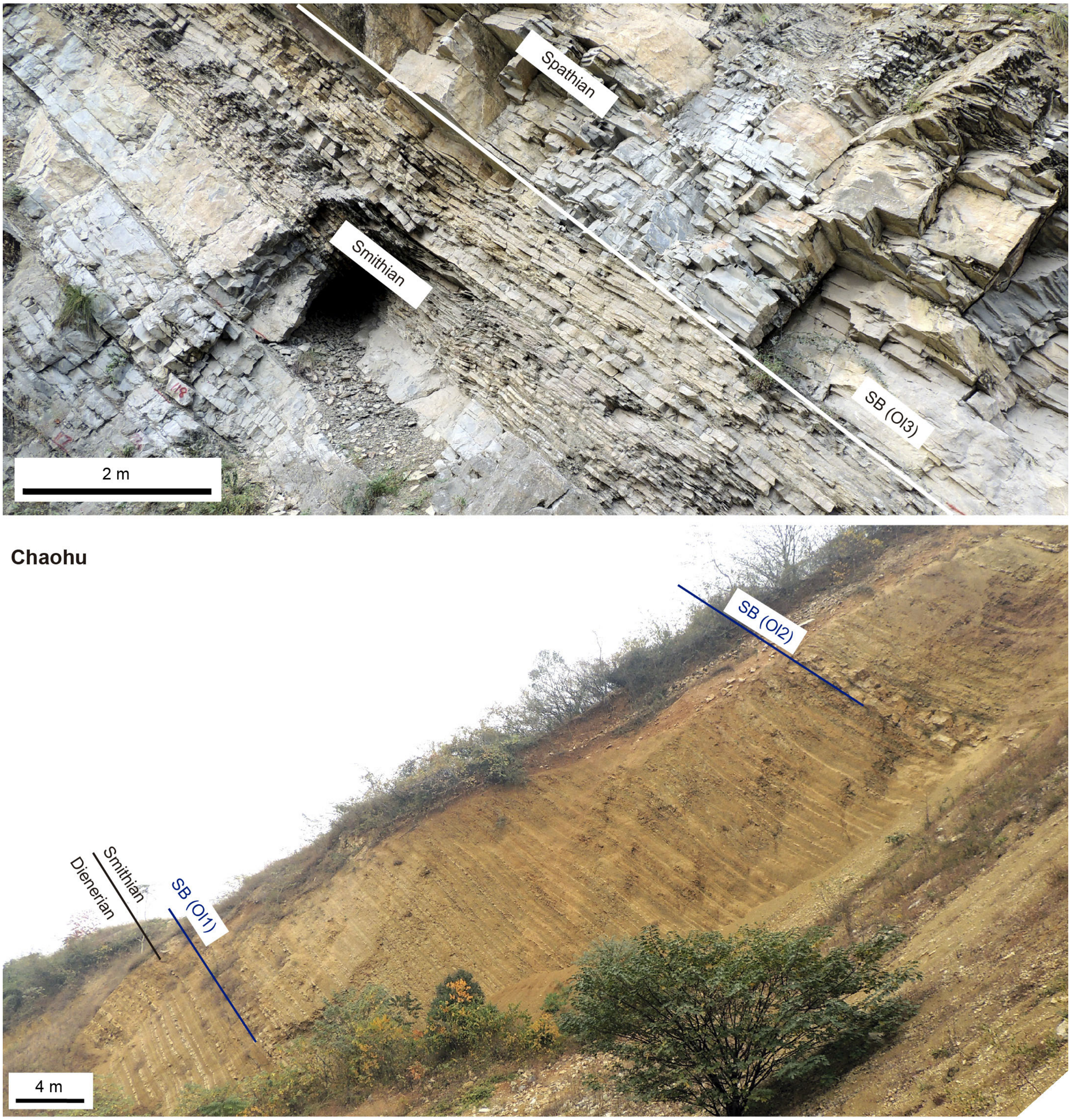

Supplementary Figure 8 Field photo and interpreted sequences of studied sections at Daxiakou and Chaohu, South China. SB = sequence boundary; OI1, OI2 and OI3 are discussed in the main text. Chronostratigraphy at both sections follows ref. 4. Photos by Mingsong Li

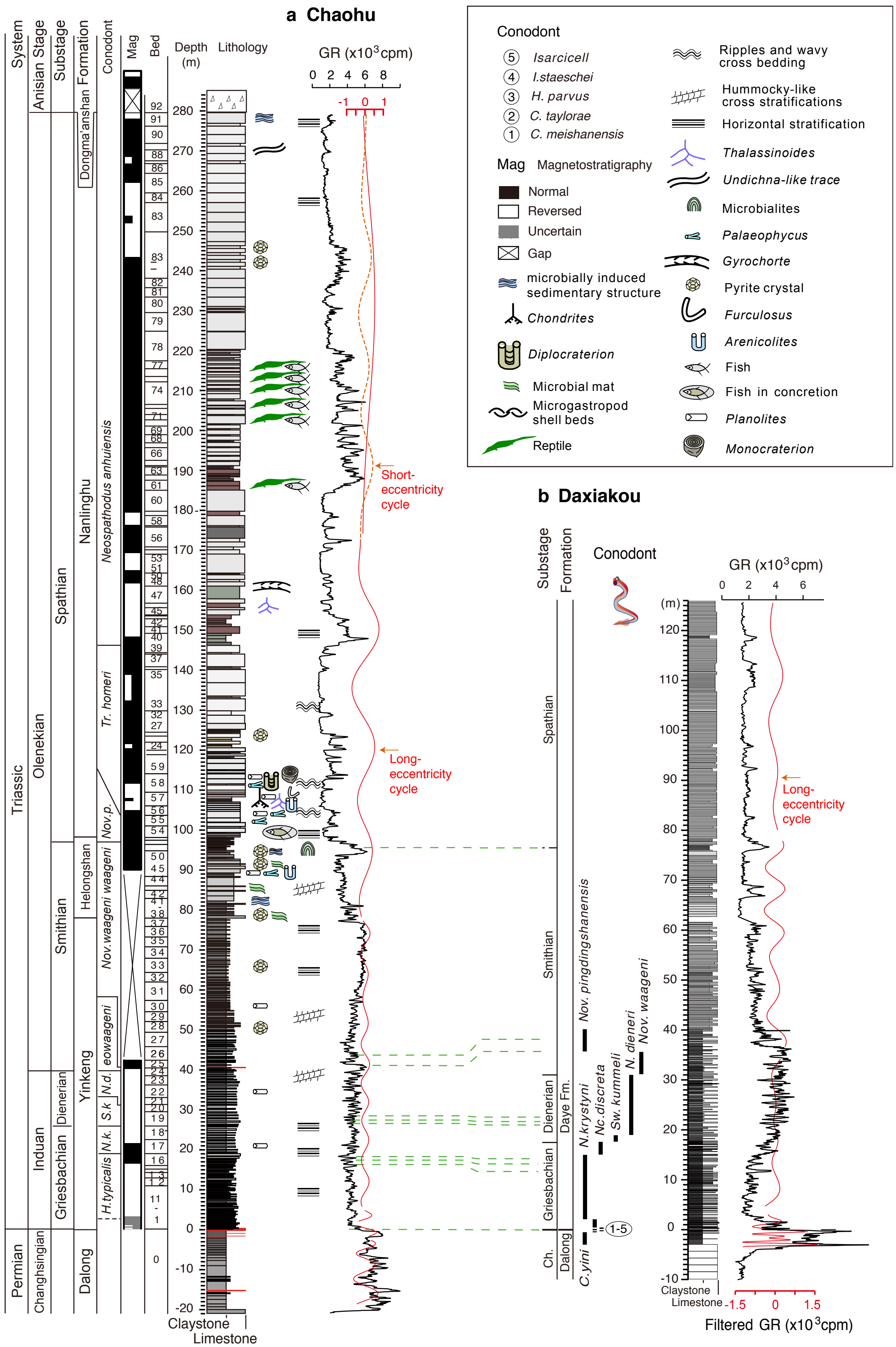

Supplementary Figure 9 Correlation of Chaohu and Daxiakou sections with the gamma ray (GR) logs at each section [4]. Biostratigraphy is according to refs. 5,6. Substage boundaries are according to refs. 4-6. Paleomagnetic polarity patterns at Chaohu are according to refs. 4,7. Filtered GR (red) and interpreted eccentricity cycles are from ref. 4. Trace fossil data are from ref. 8

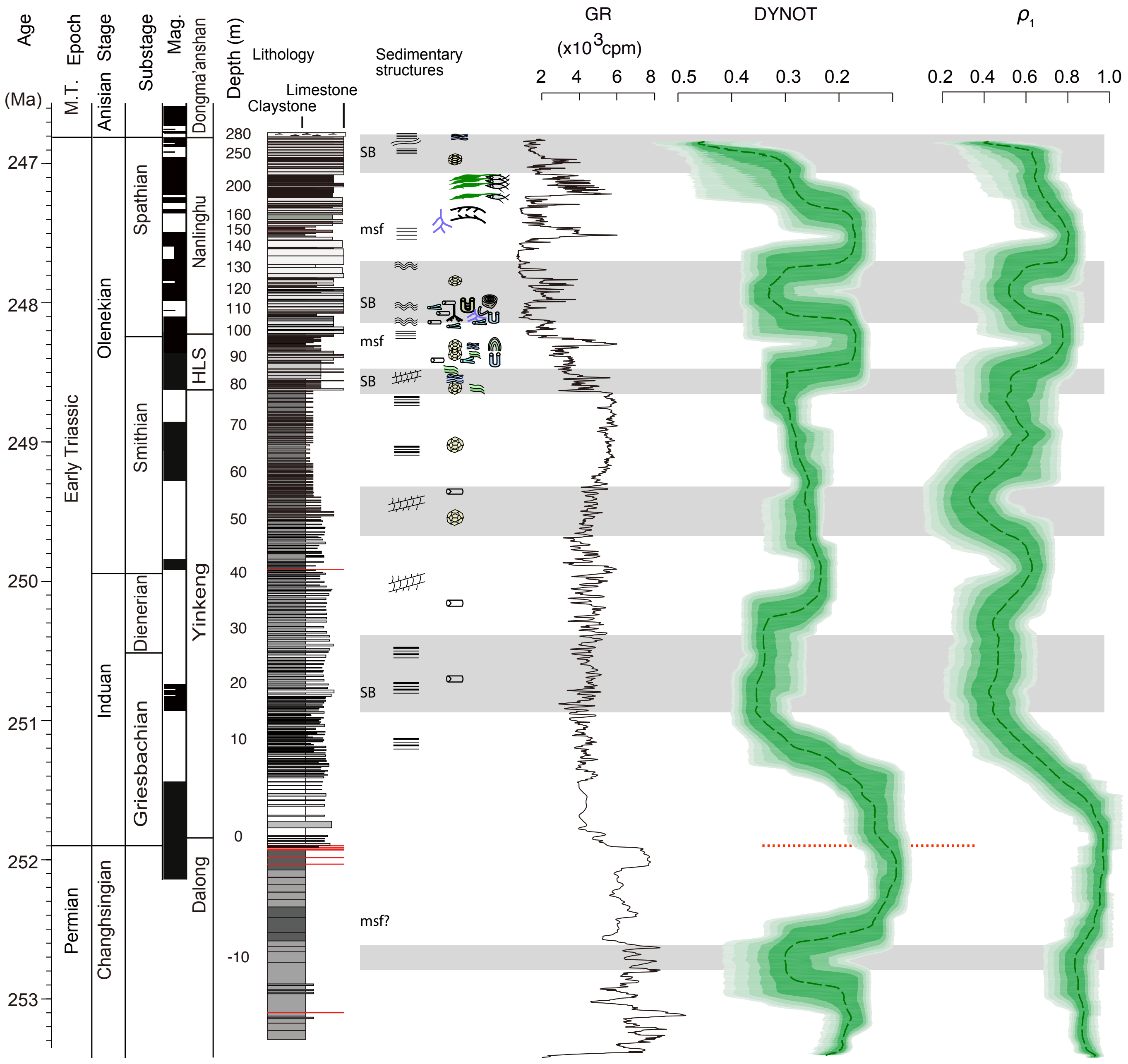

Supplementary Figure 10 Tuned gamma ray (GR) logs from the Chaohu section [4]. Biostratigraphy is according to refs. [5,6]. Substages boundaries are according to refs. 4-6. Paleomagnetic polarity patterns at Chaohu are according to refs. 4,7. Trace fossil data are from ref. 8. The DYNOT and ρ_1 models are from Fig. 6 in the main text. See Supplementary Figure 9 for legend

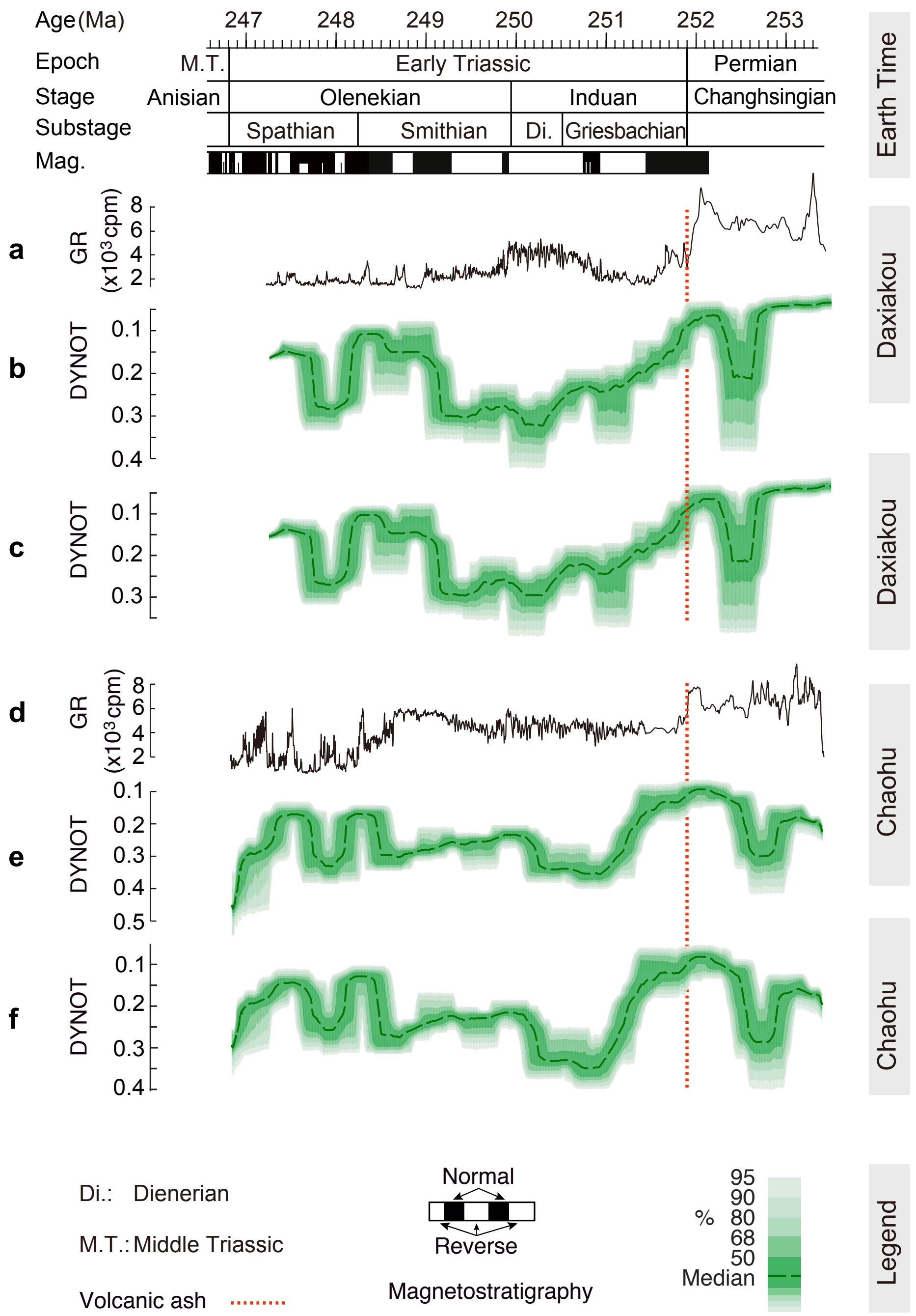

Supplementary Figure 11 DYNOT models of the Early Triassic series over different spectral bands show only minor difference at Daxiakou (**a-c**) and Chaohu (**d-f**) sections. The 0.8 to 3.0 kyr median sampling rates of the studied sections significantly limit the presence of sub-Milankovitch frequencies, thus are expected to contribute to only a minor portion of the noise. Time scale and magnetostratigraphy are from ref. 4. **a** Tuned gamma ray time series at Daxiakou [4]. **b-c** DYNOT models of (**a**) with total variance calculated over a spectral band from 1×10^3 to 1×10^6 year (**b**) and alternatively from 1.2×10^4 to 1×10^6 year (**c**). **d** Tuned gamma ray time series at Chaohu [4]. **e-f** DYNOT model of (**d**) with total variance calculated over a spectral band from 1×10^3 to 1×10^6 year (**e**) and alternatively from 1.2×10^4 to 1×10^6 year (**f**). The DYNOT models were estimated using interpolated series with random sample rates from 1.6 to 5.4 kyr for the Daxiakou gamma ray time series and 0.4 to 5.5 kyr for the Chaohu gamma ray time series, and a running window of 400 kyr. Confidence levels were estimated by a Monte Carlo analysis with 5,000 iterations

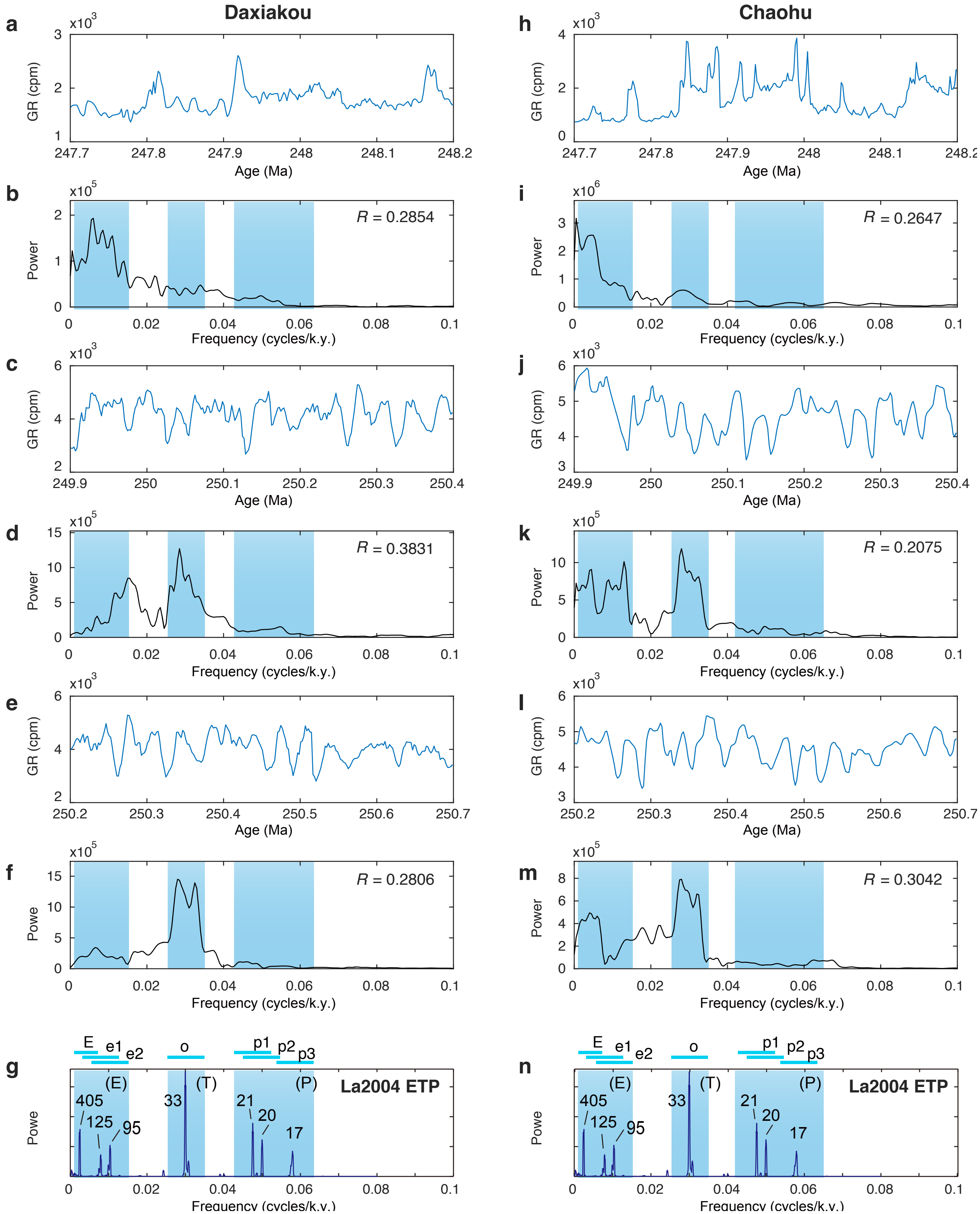

Supplementary Figure 12 Non-orbital signal (*R*) and 2π multi-taper power spectra of selected 500-kyr long intervals of the gamma-ray (GR) series from the Daxiakou (**a-f**) and Chaohu (**h-m**) sections. **g** and **n**, 2π multi-taper power spectrum of Laskar2004 ETP for 239-249 Ma. GR series are uniformly interpolated to a 2-kyr sampling rate. Each power spectrum of the GR series is plotted against the united E, T and P bands (blue bars, fixed passbands are target frequencies ± 120% × bandwidth), together with non-orbital signal ratios

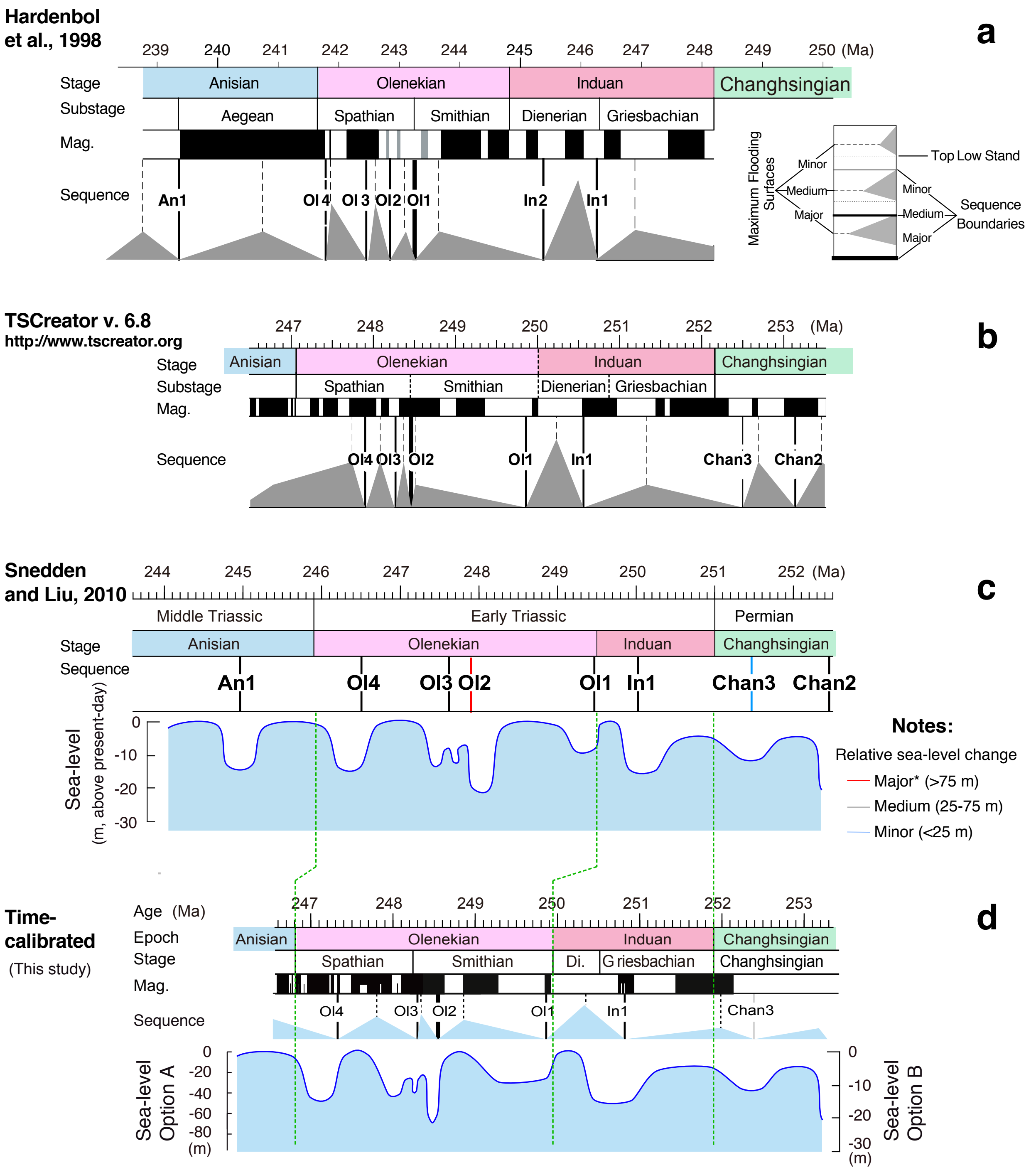

Supplementary Figure 13 Early Triassic sea-level reconstructions suggest similar sequence patterns at different timescales. **a** Timescale of European sequences during the Early Triassic anchored to the magnetostratigraphy of Arctic stratotypes [9]. **b** Sequences in the Tethyan region [10] with magnetostratigraphy of the Germanic Basin and calibrated to the age model in ref. 11. **c** Sequences and estimated sea-level change for the latest Permian-Middle Triassic [12]. There is a discrepancy in the estimated amplitude of sea-level change for Ol2, which shows sea-level change <25 m while the amplitude for Ol2 presumably can be up to >75 m. **d** Time-calibrated sea-level curve from (**c**) using the most recent age model from ref. 4. Sea-level amplitudes are provided according to Option A and Option B (see Supplementary Note 2). Mag. =Magnetostratigraphy. Dashed green lines in (**c**) and (**d**) indicate stage boundaries used for calibrating sequence boundaries

Supplementary Table 1 The 16 parameters for uncertainty analysis. Note: *bw* represents bandwidth (Methods)

Parameters	Distribution	Minimum	Maximum	Data	Plots
Sampling rate (kyr)	Fixed	1		La2004 ETP	Fig. 1; Supplementary Fig.1
		1		Site 1119	Supplementary Fig. 3**g**
		4.08			Supplementary Fig. 3**m**
		5		$\delta^{18}O$ sea-level	Supplementary Fig. 6
	Weibull & Uniform	0.20	1.55	Site 1119 original	Supplementary Fig. 5**b**
		2.04	4.08	Site 1119 original & fine tuned	Fig. 3**d**; Supplementary Fig. 3**h-l**; Supplementary Fig.5**c,g**; Supplementary Fig. 7
		0.22	2.04	Site 1119 fine-tuned	Fig. 3**c**; Supplementary Fig. 3**b-f**; Supplementary Fig. 5**f**
		0.4	5.5	Chaohu	Fig. 5**d**; Supplementary Fig. 10; Supplementary Fig. 11**e-f**
		5.5	8.25		Fig. 5**e**; Supplementary Fig. 10
		1.6	5.4	Daxiakou	Fig. 5**b**; Supplementary 11**b-c**
		5.4	8.1		Fig. 5**c**
Running window (kyr)	Uniform	300	500	La2004 ETP; ODP Site 1119	Supplementary Fig. 1**e,g,j,l**; Supplementary Fig. 3**f,l**
	Fixed	100		ODP Site 1119	Supplementary Fig. 7
		400		All series	All other figures
Cutoff frequencies in equations 1-4 (kyr^{-1}): *c1*	Uniform	1/405 - 120% × *bw*	1/405 - 90% × *bw*	All series	All DYNOT plots except Supplementary Fig. 3**e**
c2		1/405 + 90% × *bw*	1/405 + 120% × *bw*		
c3		1/125 - 120% × *bw*	1/125 - 90% × *bw*		
c4		1/125 + 90% × *bw*	1/125 + 120% × *bw*		
c5		1/95 - 120% × *bw*	1/95 - 90% × *bw*		
c6		1/95 + 90% × *bw*	1/95 + 120% × *bw*		
c7		1/40.9 - 120% × *bw*	1/40.9 - 90% × *bw*	La2004 ETP; ODP Site 1119	
		1/33 - 120% × *bw*	1/33 - 90% × *bw*	Chaohu; Daxiakou	
c8		1/40.9 + 90% × *bw*	1/40.9 + 120% × *bw*	La2004 ETP; ODP Site 1119	
		1/33 + 90% × *bw*	1/33+ 120% × *bw*	Chaohu; Daxiakou	
c9		1/23.6 - 120% × *bw*	1/23.6 - 90% × *bw*	La2004 ETP; ODP Site 1119	
		1/21 - 120% × *bw*	1/21 - 90% × *bw*	Chaohu; Daxiakou	
c10		1/23.6 + 90% × *bw*	1/23.6 + 120% × *bw*	La2004 ETP; ODP Site 1119	
		1/21 + 90% × *bw*	1/21+ 120% × *bw*	Chaohu; Daxiakou	
c11		1/22.3 - 120% × *bw*	1/22.3 - 90% × *bw*	La2004 ETP; ODP Site 1119	
		1/20 - 120% × *bw*	1/20 - 90% × *bw*	Chaohu; Daxiakou	
c12		1/22.3 + 90% × *bw*	1/22.3 + 120% × *bw*	La2004 ETP; ODP Site 1119	
		1/20 + 90% × *bw*	1/20+ 120% × *bw*	Chaohu; Daxiakou	
c13		1/19.1 - 120% × *bw*	1/19.1 - 90% × *bw*	La2004 ETP; ODP Site 1119	
		1/17 - 120% × *bw*	1/17 - 90% × *bw*	Chaohu; Daxiakou	
c14		1/19.1 + 90% × *bw*	1/19.1 + 120% × *bw*	La2004 ETP; ODP Site 1119	
		1/17 + 90% × *bw*	1/17+ 120% × *bw*	Chaohu; Daxiakou	

Supplementary Table 2 Sampling rate for each of the studied time series. Unit is kyr

Data	Mean sampling rate	Median sampling rate	5th percentile	95th percentile
Original ODP Site 1119 gamma ray series	0.87	0.82	0.20	1.55
Fine-tuned ODP Site 1119 gamma ray series	0.87	0.85	0.22	2.04
Tuned Chaohu gamma ray series	2.18	1.60	0.40	5.50
Full tuned Daxiakou gamma ray series	4.06	3.00	1.60	9.90
Triassic tuned Daxiakou gamma ray series	3.16	2.90	1.60	5.40

Supplementary Table 3 Age model for fine-tuning of the gamma-ray series at ODP Site 1119. The age model is based on filtered obliquity cycles in Supplementary Fig. 4. "Original age" is age in refs. 1,13. "New age" is age used in this study. Unit is ka (kiloyears before present)

Original age	New age	Original age	New age	Original age	New age	Original age	New age
26.5	26.5	398.9	394.6	766.0	762.7	1140.0	1130.8
65.9	67.4	437.5	435.5	804.0	803.6	1178.0	1171.7
107.0	108.3	475.3	476.4	834.5	844.5	1213.0	1212.6
147.3	149.2	514.8	517.3	875.6	885.4	1258.7	1253.5
185.1	190.1	550.9	558.2	924.1	926.3	1306.0	1294.4
227.0	231.0	588.7	599.1	966.9	967.2	1346.0	1335.3
270.0	271.9	632.0	640.0	1018.0	1008.1	1387.0	1376.2
310.9	312.8	664.4	680.9	1044.0	1049.0		
356.1	353.7	720.3	721.8	1103.0	1089.9		

Supplementary Note 1. Contributing factors to the sedimentary noise

The sedimentary noise model is designed to extract water-depth related noise as an indicator for relative sea-level change. However, other factors may also contribute to “noise” estimated by the DYNOT and ρ_1 models, as follows.

Basin-scale tectonic subsidence changes not related to orbital forcing may affect long-term relative sea-level change [14-17] and influence the DYNOT and ρ_1 models. There are also potential effects from earthquakes, which could impose an episodic, 10^4 year recurrence interval on passive margins [18] disturbances within a sea level proxy series, elevating noise at all frequencies and/or amplifying earthquake recurrence frequencies.

South China was an isolated island during the Permian according to paleomagnetic studies from the South China and North China plates [19-21]. Paleomagnetic results indicated that the South China plate rotated ~70° clockwise during the Triassic when it was accreted to the North China plate, causing the collision to progress from east to west after the early Triassic Period [20]. The collision presumably persisted until the late Triassic, because the two blocks started to share the same paleomagnetic pole in the Jurassic [19,22] that suggests the collision took place prior to the Jurassic [23]. The collision of the North China and South China plates led to gradually uplift and shallowing of the marginal basin of the northern South China plate during the Early-Middle Triassic. These tectonic processes may have impacts on the long-term trend of sedimentary noise, and cannot be removed from the two models in this study.

Paleoclimate proxy records of orbital forcing are fundamentally non-linear [24-26]. For example, Ripepe and Fischer [27] modeled the combined effect of non-linear accumulation and bioturbation on precession-forced basinal carbonate cycles to demonstrate how the modulation of the precession index, namely, the eccentricity, is magnified. With each step, more variance is transferred from the precession into the eccentricity band. The final spectrum is dominated by eccentricity, while the power in the precession band is significantly reduced. Thus, the presence of eccentricity in paleoclimatic spectra can be explained by non-linear or rectifying responses of the climate and/or depositional system to orbital forcing [27]. The effect on the sedimentary noise model in the case when variance is transferred from one orbital band to another is that the filtering will still remove the orbital signal but now from another orbital band. In the event of sideband tone generation [28] some orbital signal may not be captured and removed. However, the recommended filter bandwidths for the DYNOT model are extremely generous (see Fig. 1**c**, and main text), and should be sufficient to attenuate most nonlinearities related to orbital forcing. On the other hand, non-linear climate responses can lead to higher ρ_1 values, which has yet to be explored.

Sub-Milankovitch cycles are kyr-scale climate cycles with significant amplitudes in ultra-high resolution paleoclimatic records [29-32]. Sub-Milankovitch scale climate variability is usually of lower amplitude than Milankovitch band climate variability [33]. The power spectrum of climate variability has a rapid decrease of power with increasing frequency due to a low pass filtering effect [34-36] and from non-linear responses (see above). Sub-Milankovitch cycles at the Chinese sections contribute to only a minor portion of the sedimentary noise (Supplementary Fig. 11).

Volcanisms may have multiple influences on the sedimentary noise model. Firstly, it may alter proxy records, and distort the noise spectrum near volcanic ash beds. For example, ash beds near the Permian-Triassic boundary in the marine sections at Meishan, Chaohu and Daxiakou in South China are accompanied by elevated levels of thorium and potassium [4], which lead to non-climatic high amplitude peaks in the gamma ray series. Secondly, high volume volcanism may lead to a series of climate changes that drive hydrological cycles not paced by Milankovitch cycles. Volcanic ash beds highlighted with dashed red lines in Fig. 6 near the Permian-Triassic boundary at Chaohu and Daxiakou likely contribute to low DYNOT and high ρ_1 values.

Decimeter-scale marine carbonate-shale bedding represents a combination of primary deposition and post-depositional processes [37-40]. Diagenetic segregation is thought to transfer carbonate from carbonate-poor to adjacent carbonate-rich layers, leading to relative enhancement of orbital signals. If this differential diagenesis significantly distorts the primary paleoclimate signal or generates new rhythms [26,41], noise contributions to the DYNOT and ρ_1 models may increase.

The diagenetic alteration of studied Chinese sections is negligible. Conodont color indicative of alteration thermal alteration of conodont fossils [42] is pale brown at Daxiakou [6], indicating no significant diagenesis in the Upper Permian to Lower Triassic at Daxiakou. At Chaohu, the lack of correlation between $\delta^{18}O_{carb}$, $\delta^{13}C_{carb}$, carbonate content, and $\delta^{13}C_{carb}$, and coupling of $\delta^{13}C_{carb}$ and $\delta^{13}C_{org}$ indicate that minimal diagenetic alteration occurred in the section [43].

Supplementary Note 2. Main reference scale for Early Triassic sea-level variations

In 1998, as an appendix to a special volume on the Mesozoic and Cenozoic sequence stratigraphy of European basins [44], a team at Exxon led by Jan Hardenbol produced a suite of charts summarizing Mesozoic-Cenozoic biostratigraphic correlations and placement of presumed global sequences and magnitude of associated sea-level changes [9] that was a major update and revision of the previous "Exxon" sea-level scale [45]. The details on the recognition and biostratigraphic calibration of these sequences and derivation of their magnitudes were never published, but important reference scales for the Triassic Period were compilations from the Italian Dolomites [46] and Canadian Arctic [47]. The age control on these Dolomites and Canadian Arctic reference sections has been significantly enhanced since this 1998 publication through applications of magnetostratigraphy [48-50], of additional conodont and ammonoid stratigraphy [51,52], and of carbon-isotope stratigraphy [53,54]. However, the basic sequence-stratigraphy scale for the Early Triassic has remained stable [12,55].

The amplitude of sea-level variations during the Early Triassic [12] points to two options (Supplementary Fig. 13**d**): Option A: sea-level changes were up to 75+ m as explained below; and Option B: plot of sea-level curve in ref. 12 indicate sea-level changes that were <25 m. Sea-level falls defined by the third order sequence boundaries were interpreted to be <25 m for a minor relative sea-level change, 25-75 m for a medium relative sea-level change, and >75 m for a major relative sea-level change [12]. The amplitude of all relative sea-level changes in the Early Triassic is assigned to the "medium" level except the second sequence boundary in the Olenekian, Ol2, which has been considered to represent a major sea-level fall [12].

Most of the Early Triassic sequences in ref. 12 were recognized in the Boreal and Tethyan provinces of the European basins, Arctic Canada, Pakistan, and other regions [9,45-47,56,57]. Deep basin to slope environments for the Lower Triassic at Chaohu in South China are discussed in ref. 58. The Early Triassic depositional environment at Chaohu has been interpreted to be open-marine basin with a depth of a few hundred meters [59]. Thick-bedded limestone with hummocky cross-stratification in the overlying Helongshan Formation (late Smithian) represents storm deposits on a continental slope [60]. The presence of thick-bedded limestone with hummocky cross-stratification in the upper Nanlinghu Formation (late Spathian) indicates episodically high energy environments [61]. This evidence suggests an amplitude of sea-level change at the 75+ m scale of Option A, rather than <25 m of Option B.

Supplementary Note 3. Obliquity forcing and land-ocean water exchange

Early Triassic paleogeographic reconstructions (e.g., Fig. 4 in the main text) shows that vast areas of the Pangea Supercontinent were located in middle-high latitude regions, as well as in low latitude areas such as the Germanic Basin, serving as major aquifers, and potential key regions for obliquity-forced water storage. Lower

water storage in the Germanic Basin interpreted from continental sequence boundaries [62,63] correlates with decreased obliquity forcing in South China and high global sea level (Fig. 6 in the main text). Our interpretation of these relationships is that obliquity forcing alters meridional heat transport, thus the flux of water vapor from ocean to land. This affects lake and aquifer water storage, and consequently, sea-level [35].

Late Cenozoic paleoclimate studies have proposed that obliquity forcing dominated the poleward flux of heat, moisture, and precipitation through the control of differential insolation between high and low latitude [64,65]. For example, the difference in summer half-year insolation between 25° N and 70º N is almost completely dominated by obliquity [64]; this difference was proposed to drive the meridional flux of heat, water vapor, and precipitation. Similar obliquity forcing mechanisms would be expected to be in place during the greenhouses of the Early Triassic [35] and mid-Cretaceous [66]. The obliquity forcing extends to the long-period modulation of the obliquity: the 1.2-myr obliquity nodes would be associated with reduced poleward transportation of heat, moisture, and precipitation, and the 1.2-myr obliquity variation maxima would be linked to invigorated heat and moisture transportation and intensified precipitation [35,66]. In the Early Triassic, the observed obliquity intervals in low-latitude marine sections from South China correlate to 1.2-myr obliquity modulation cycles of extended theoretical astronomical solutions, suggesting a causal link between million-year scale obliquity forcing and low-latitude climate change [35].

Alternative mechanisms of obliquity forcing have also been proposed: Global water storage on continents in opposing northern and southern hemisphere latitudes canceled precession and reinforced obliquity-forced responses (e.g., ref. 67); climate change may also have been sensitive to annual average insolation [68] or integrated summer energy [69], which is also controlled by obliquity.

Supplementary References

1. Carter, R.M. & Gammon, P. New Zealand Maritime Glaciation: Millennial-scale southern climate change since 3.9 Ma. *Science* **304**, 1659-1662 (2004).
2. Miller, K.G. *et al.* The Phanerozoic record of global sea-level change. *Science* **310**, 1293-1298 (2005).
3. Meyers, S.R. & Hinnov, L.A. Northern Hemisphere glaciation and the evolution of Plio-Pleistocene climate noise. *Paleoceanography* **25**, PA3207, https://doi.org/10.1029/2009PA001834 (2010).
4. Li, M. *et al.* Astronomical-cycle scaling of the end-Permian extinction and the Early Triassic Epoch of South China and Germany. *Earth Planet. Sci. Lett.* **441**, 10-25 (2016).
5. Zhao, L. *et al.* Lower Triassic conodont sequence in Chaohu, Anhui Province, China and its global correlation. *Palaeogeogr. Palaeoclimatol. Palaeoecol.* ***252**, 24-38 (2007).*
6. Zhao, L., Chen, Y. & Chen, Z.Q. Uppermost Permian to lower Triassic conodont zonation from Three George area, South China. *Palaios* **28**, 509-522 (2013).
7. Sun, Z. *et al.* Magnetostratigraphy of the Lower Triassic beds from Chaohu (China) and its implications for the Induan–Olenekian stage boundary. *Earth Planet. Sci. Lett.* **279**, 350-361 (2009).
8. Chen, Z.-Q., Tong, J. & Fraiser, M.L. Trace fossil evidence for restoration of marine ecosystems following the end-Permian mass extinction in the Lower Yangtze region, South China. *Palaeogeogr. Palaeoclimatol. Palaeoecol.* **299**, 449-474 (2011).
9. Hardenbol, J. *et al.* Mesozoic and Cenozoic sequence chronostratigraphic framework of European basins. in *Mesozoic and Cenozoic Sequence Stratigraphy of European Basins*, **60**, 3-13 (Soc. Sediment. Geol. Spec. Publ., 1998).
10. Ogg, J. & Lugowski, A. TS creator–visualization of enhanced Geologic Time Scale 2012 database. (2013).
11. Gradstein, F., Ogg, J., Schmitz, M. & Ogg, G. (eds.). *The Geological Time Scale 2012*, 1-1144 (Elsevier, 2012).
12. Snedden, J. & Liu, C. A compilation of Phanerozoic sea-level change, coastal onlaps and recommended sequence designations. *AAPG Search and Discovery Article* **40594**, http://www.searchanddiscovery.com/pdfz/documents/2010/40594snedden/ndx_snedden.pdf.html (2010).
13. Carter, R.M., Gammon, P.R. & Millwood, L. Glacial–interglacial (MIS 1–10) migrations of the Subtropical Front across ODP Site 1119, Canterbury Bight, Southwest Pacific Ocean. *Mar. Geol.* **205**, 29-58 (2004).
14. Haq, B.U. & Schutter, S.R. A chronology of Paleozoic sea-level changes. *Science* **322**, 64-68 (2008).
15. Catuneanu, O. *Principles of Sequence Stratigraphy*, 1-375 (Elsevier, 2006).
16. Catuneanu, O. *et al.* Towards the standardization of sequence stratigraphy. *Earth-Sci. Rev.* **92**, 1-33 (2009).

17. Müller, R.D., Sdrolias, M., Gaina, C., Steinberger, B. & Heine, C. Long-term sea-level fluctuations driven by ocean basin dynamics. *Science* **319**, 1357-1362 (2008).
18. Talwani, P. & Schaeffer, W.T. Recurrence rates of large earthquakes in the South Carolina Coastal Plain based on paleoliquefaction data. *J. Geophys. Res. Solid Earth* **106**, 6621-6642 (2001).
19. Lin, J., Fuller, M. & Zhang, W. Preliminary Phanerozoic Polar Wander Paths for the North and South China Blocks. *Nature* **313**, 444-449 (1985).
20. Zhao, X. & Coe, R.S. Paleomagnetic constraints on the collision and rotation of North and South China. *Nature* **327**, 141-144 (1987).
21. McElhinny, M.W., Embleton, B.J.J., Ma, X.H. & Zhang, Z.K. Fragmentation of Asia in the Permian. *Nature* **293**, 212-216 (1981).
22. Enkin, R.J., Yang, Z., Chen, Y. & Courtillot, V. Paleomagnetic constraints on the geodynamic history of the major blocks of China from the Permian to the present. *J. Geophys. Res.* **97**, 13953-13989 (1992).
23. Meng, Q.-R. & Zhang, G.-W. Timing of collision of the North and South China blocks: Controversy and reconciliation. *Geology* **27**, 123-126 (1999).
24. Weedon, G.P. *Time series analysis and cyclostratigraphy: Examining stratigraphic records of environmental cycles*, 259 (Cambridge University Press, 2003).
25. Hinnov, L.A. Cyclostratigraphy and its revolutionizing applications in the earth and planetary sciences. *Geol. Soc. Am. Bull.* **125**, 1703-1734 (2013).
26. Hilgen, F.J. *et al.* Stratigraphic continuity and fragmentary sedimentation: the success of cyclostratigraphy as part of integrated stratigraphy. *Geol. Soc. London Spec. Publ.* **404**, 157-197 (2015).
27. Ripepe, M. & Fischer, A.G. Stratigraphic rhythms synthesized from orbital variations. *J. Sediment. Petrol.* **63**, 335-344 (1991).
28. Rial, J.A. & Anaclerio, C. Understanding nonlinear responses of the climate system to orbital forcing. *Quat. Sci. Rev.* **19**, 1709-1722 (2000).
29. Elrick, M. & Hinnov, L.A. Millennial-scale climate origins for stratification in Cambrian and Devonian deep-water rhythmites, western USA. *Palaeogeogr. Palaeoclimatol. Palaeoecol.* **123**, 353-372 (1996).
30. Soon, W. *et al.* A review of Holocene solar-linked climatic variation on centennial to millennial timescales: Physical processes, interpretative frameworks and a new multiple cross-wavelet transform algorithm. *Earth-Sci. Rev.* **134**, 1-15 (2014).
31. Franco, D.R., Hinnov, L.A. & Ernesto, M. Millennial-scale climate cycles in Permian-Carboniferous rhythmites: Permanent feature throughout geologic time? *Geology* **40**, 19-22 (2012).
32. Dansgaard, W. *et al.* Evidence for general instability of past climate from a 250-kyr ice-core record. *Nature* **364**, 218-220 (1993).
33. Hagelberg, T.K., Bond, G. & Demenocal, P. Milankovitch band forcing of sub-Milankovitch climate variability during the Pleistocene. *Paleoceanography* **9**, 545-558 (1994).
34. Hays, J.D., Imbrie, J. & Shackleton, N.J. Variations in the Earth's Orbit: Pacemaker of the Ice Ages. *Science* **194**, 1121-1132 (1976).
35. Li, M. *et al.* Obliquity-forced climate during the Early Triassic hothouse in China. *Geology* **44**, 623-626 (2016).
36. Mann, M.E. & Lees, J.M. Robust estimation of background noise and signal detection in climatic time series. *Clim. Change* **33**, 409-445 (1996).
37. Hallam, A. Origin of the limestone-shale rhythm in the Blue Lias of England: A composite theory. *J. Geol.* **72**, 157-169 (1964).
38. Hallam, A. Origin of minor limestone-shale cycles: Climatically induced or diagenetic? *Geology* **14**, 609-612 (1986).
39. Weedon, G.P., Jenkyns, H.C., Coe, A.L. & Hesselbo, S.P. Astronomical calibration of the Jurassic time-scale from cyclostratigraphy in British mudrock formations. *Phil. Trans. R. Soc.* **357**, 1787-1813 (1999).
40. Bottjer, D.J., Arthur, M.A., Dean, W.E., Hattin, D.E. & Savrda, C.E. Rhythmic bedding produced in Cretaceous pelagic carbonate environments: sensitive recorders of climatic cycles. *Paleoceanography* **1**, 467-481 (1986).
41. Westphal, H., Hilgen, F. & Munnecke, A. An assessment of the suitability of individual rhythmic carbonate successions for astrochronological application. *Earth-Sci. Rev.* **99**, 19-30 (2010).
42. Epstein, A.G., Epstein, J.B. & Harris, L.D. Conodont color alteration: an index to organic metamorphism. *U.S. Geol. Surv. Prof. Pap.* **995**, 1-27 (1977).
43. Fu, W. *et al.* Eccentricity and obliquity paced carbon cycling in the Early Triassic and implications for post-extinction ecosystem recovery. *Sci. Rep.* **6**, 27793, https://doi.org/10.1038/srep27793 (2016).
44. Graciansky, P., Hardenbol, J., Jaquin, T. & Vail, P.R. (eds.). *Mesozoic and Cenozoic Sequence Stratigraphy of European Basins*, 786 (Soc. Sediment. Geol. Spec. Publ., 1998).

45. Haq, B.U., Hardenbol, J. & Vail, P.R. Chronology of fluctuating sea levels since the Triassic. *Science* **235**, 1156-1167 (1987).
46. Gianolla, P. & Jacquin, T. Triassic sequence stratigraphic framework of western European basins. in *Mesozoic and Cenozoic Sequence Stratigraphy of European Basins*, **60**, 643-650 (Soc. Sediment. Geol. Spec. Publ., 1998).
47. Embry, A.F. Global sequence boundaries of the Triassic and their identification in the Western Canada Sedimentary Basin. *Bull. Can. Pet. Geol.* **45**, 415-433 (1997).
48. Graziano, S. & Ogg, J. Lower Triassic magnetostratigraphy in the Dolomites region (Italy) and correlation to Arctic ammonite zones. (AGU Fall Meeting, 1994).
49. Scholger, R., Mauritsch, H.J. & Brandner, R. Permian–Triassic boundary magnetostratigraphy from the southern Alps (Italy). *Earth Planet. Sci. Lett.* **176**, 495-508 (2000).
50. Hounslow, M.W. & Muttoni, G. The geomagnetic polarity timescale for the Triassic: linkage to stage boundary definitions. in *The Triassic Timescale*, (ed. Lucas, S.G.) 61-102 (Geol. Soc. London, 2010).
51. Baud, A. *et al.* Lower Triassic bryozoan beds from Ellesmere Island, High Arctic, Canada. *Polar Res.* **27**, 428-440 (2008).
52. Posenato, R. Global correlations of mid Early Triassic events: The Induan/Olenekian boundary in the Dolomites (Italy). *Earth-Sci. Rev.* **91**, 93-105 (2008).
53. Grasby, S.E., Beauchamp, B., Embry, A. & Sanei, H. Recurrent Early Triassic ocean anoxia. *Geology* **41**, 175-178 (2013).
54. Foster, W.J., Danise, S., Price, G.D. & Twitchett, R.J. Subsequent biotic crises delayed marine recovery following the late Permian mass extinction event in northern Italy. *PloS ONE* **12**, e0172321, https://doi.org/10.1371/journal.pone.0172321 (2017).
55. Snedden, J.W. & Liu, C. Recommendations for a uniform chronostratigraphic designation system for Phanerozoic depositional sequences. *AAPG bull.* **95**, 1095-1122 (2011).
56. Al-Husseini, M.I. & Koehrer, B. Chrono- and sequence-stratigraphy of the Mid-Permian to Early Triassic Khuff sequences of the Arabian Plate. *GeoArabia* **18**, 103-130 (2013).
57. Hermann, E. *et al.* Organic matter and palaeoenvironmental signals during the Early Triassic biotic recovery: The Salt Range and Surghar Range records. *Sediment. Geol.* **234**, 19-41 (2011).
58. Li, S. & Wu, S. Study on petrology and sedimentary environments of the Qinglong Group of the Lower–Middle Triassic in Chaoxian, Anhui. in *Study on Lithofacies Paleogeography of Qinglong Group of Lower–Middle Triassic in the Lower Yangtze River Region* (ed. Feng, Z.) 82-92 (Yunnan Sci. Tech. Press, Kunming, 1988).
59. Han, S. Preliminary study on origin of the Lower Triassic nodular limestone in the region along the Yangtze river. *Chin. J. Geol.* **3**, 232–238 (1983).
60. Tong, J. Lower Triassic sequence stratigraphy of Chaoxian, Anhui. *Bull. Chin. Acad. Geol. Sci.* **18**, 215–219 (1997).
61. Li, S., Tong, J., Liu, K., Wang, F. & Huo, Y. The Lower Triassic cyclic deposition in Chaohu, Anhui Province, China. *Palaeogeogr. Palaeoclimatol. Palaeoecol.* **252**, 188-199 (2007).
62. Bourquin, S., Durand, M., Diez, J., Broutin, J. & Fluteau, F. The Permian-Triassic boundary and Early Triassic sedimentation in Western European basins: an overview. *J. Iber. Geol.* **33**, 221-236 (2007).
63. Aigner, T. & Bachmann, G.H. Sequence-stratigraphic framework of the German Triassic. *Sediment. Geol.* **80**, 115-135 (1992).
64. Raymo, M.E. & Nisancioglu, K. The 41 kyr world: Milankovitch's other unsolved mystery. *Paleoceanography* **18**, 1011, https://doi.org/10.1029/2002PA000791 (2003).
65. Young, M.A. & Bradley, R.S. Insolation Gradients and the Paleoclimatic Record. in *Milankovitch and Climate*, **126** (eds. Berger, A., Imbrie, J., Hays, J., Kukla, G. & Saltzman, B.) 707-713 (Springer, 1984).
66. Wendler, J.E. & Wendler, I. What drove sea-level fluctuations during the mid-Cretaceous greenhouse climate? *Palaeogeogr. Palaeoclimatol. Palaeoecol.* **441**, 412-419 (2016).
67. Hinnov, L. & Park, J.J. Strategies for assessing Early-Middle (Pliensbachian-Aalenian) Jurassic cyclochronologies. *Phil. Trans. R. Soc.* **357**, 1831-1859 (1999).
68. Naish, T. *et al.* Obliquity-paced Pliocene West Antarctic ice sheet oscillations. *Nature* **458**, 322-328 (2009).
69. Huybers, P. Early Pleistocene glacial cycles and the integrated summer insolation forcing. *Science* **313**, 508-511 (2006).
